# Psychological predictors of chronic pain in Al Kharj region, Saudi Arabia

**DOI:** 10.1186/s12991-021-00345-3

**Published:** 2021-03-26

**Authors:** Jamaan Al-Zahrani, Mamdouh M. Shubair, Sameer Al-Ghamdi, Khaled K. Aldossari, Majid Alsalamah, Badr F. Al-Khateeb, Abdulkarim Saeed, Saeed Alshahrani, Aseel Salem AlSuwaidan, Abdullah A. Alrasheed, Ashraf El-Metwally

**Affiliations:** 1 Family & Community Medicine Department, College of Medicine, Prince Sattam bin Abdulaziz University, Al Kharj, 11942 Saudi Arabia; 2 School of Health Sciences, University of Northern British Columbia (UNBC), 3333 University Way, Prince George, BC V2N 4Z9 Canada; 3 Department of Emergency Medicine, King Abdul-Aziz Medical City, College of Public Health and Health Informatics (CPHHI), King Saud-Bin Abdu-Aziz for Health Sciences (KSAU-HS), Riyadh, Saudi Arabia; 4 Department of Family Medicine, King Abdul-Aziz Medical City, College of Public Health and Health Informatics (CPHHI), King Saud-Bin Abdu-Aziz for Health Sciences (KSAU-HS), Riyadh, Saudi Arabia; 5 College of Medicine, King Saud bin Abdulaziz University for Health Sciences, Riyadh, 14611 Saudi Arabia; 6 Health Programs General Department, Ministry of Health, Riyadh, Saudi Arabia; 7 Department of Family and Community Medicine, College of Medicine, King Saud University, Riyadh, Saudi Arabia; 8 College of Public Health and Health Informatics, King Saud Bin Abdulaziz University for Health Sciences, King Abdullah International Medical Research Centre, Ministry of the National Guard Health Affairs, Riyadh, Kingdom of Saudi Arabia

**Keywords:** General Health Questionnaire-12, Chronic pain, Psychological distress, Saudi Arabia

## Abstract

**Background:**

Psychological distress is one of the major determinants for the experience progression, and recovery of chronic pain. However, it is unclear whether physical pain in specific body sites could be predictive of psychological illness. In this study, we aim to investigate the link between chronic pain in specific anatomical sites and psychological distress represented in the General Health Questionnaire-12 (GHQ-12 items).

**Methods:**

A population-based cross-sectional study was conducted in Al Kharj region of Saudi Arabia. We included 1003 participants. Data were collected using the GHQ-12, and a subjective report on eight anatomical pain sites. Data analysis used statistical software SPSS version 26.0 for Windows statistical package.

**Results:**

Chronic musculoskeletal pain in the neck and head regions was significantly associated with higher psychological distress. Other sites (back, lower limb, chest, abdominal and upper limb pain) were not associated with psychological distress. In multiple regression analysis, chronic ‘general’ pain was significantly associated with higher psychological distress (unstandardized Beta regression coefficient = 2.568; *P* < 0.0001). The patients with younger age were more likely to develop negative psychological disorders (unstandardized Beta = − 3.137; *P* = 0.038). Females were more likely to have higher psychological distress than males (unstandardized Beta = 2.464, *P* = 0.003). Single (not-married) people have a higher risk of psychological distress than married people (unstandardized Beta = 2.518, *P* = 0.025). Also, job type/status whether being unemployed (not working) or ‘civilian’ (civil servant/worker) was positively and significantly associated with an increased probability of psychological distress (unstandardized Beta = 1.436, *P* = 0.019).

**Conclusion:**

Chronic ‘general’ pain was significantly associated with negative psychological disorders. The government of Saudi Arabia needs to focus on patients with chronic ‘general’ pain, females, young and unmarried individuals as potentially ‘high-risk’ population subgroups for adverse psychological disorders, and subsequent long-term complications.

## Background

Chronic pain is a subjective, unpleasant sensory and emotional experience that persists beyond average healing time, i.e., more than 3 months [1]. It is a complex and multidimensional phenomenon that is uniquely perceived by each individual suffering from it. Several factors modulate the severity and chronicity of pain, including the extent of the injury or illness, the presence of medical or psychological comorbidities, as well as environmental influences [1, 2]. The prevalence of chronic pain in United States has been observed to be 30.7% [3]. The prevalence of chronic pain increases with age and correlates with lower socioeconomic status [3]. It is also more likely to affect females than males [3].

According to the Global Burden of Disease Study 2016, chronic pain is considered the leading cause of disability worldwide, and its burden seems to be growing rapidly. Tension-type headaches affect 1.9 billion people worldwide, while the lower-back and the neck have been shown to be the most common pain sites related to years lived with disability [4]. In the United States, pain-related disability costs 261 to 300 billion dollars annually [1]. The indirectly measured costs related to decreased economic productivity have exceeded 600 billion Dollars, which is higher than what diabetes, heart diseases, and cancer would cost [5]. Chronic pain is widely associated with medical comorbidities. The proportion of patients suffering from chronic pain was 49.2% in osteoporosis, 45.2% in arthritis, 41.4% in bronchitis/COPD, 36.8 in neck/back disorder, 34.6% in stroke, and 34.3 in heart disease [6].

Besides the impact of medical comorbidities, it is evident that psychosocial factors influence the duration of pain, the progression from acute to chronic, and the recovery to functional status [7]. In fact, mental illness has a great impact on the expression of physical symptoms. The cognitive–emotional experience of pain could facilitate or inhibit the intensity processed through multiple regions in the brain and hence, modulate the perception of pain [8]. In depression, the dysfunction of the mesolimbic system in the brain impairs the motivational behavior which might alter pain severity and chronicity [8]. Also, it is well recognized that psychological disorders, including depression and anxiety, increase the risk of pain-related disability and the frequency of medical consultations [9]. The prevalence of depression in patients with chronic pain ranges from 4.7 to 22% and 5.9 to 46% in population-based studies and in primary healthcare settings, respectively [10]. A higher proportion of depression was reported in patients attending specialized chronic pain centers [11]. One study found that, in patients with chronic pain, the prevalence of depression was significantly high as compared to the healthy group [12]. Community-based survey analysis of 44 out of 47 low- and middle-income countries showed that the prevalence of depression was significantly higher in individuals who experience severe pain [13].

GHQ-12 could be used as predictive tool for pain in certain body sites [14, 15]. In Japan, participants with lower mental health statuses were more likely to experience pain in several body sites compared to their more mentally stable counterparts [14]. Higher scores of GHQ-12 could also indicate eye fatigue and poor sleeping habits [14, 16]. Subjective fatigue was associated with eye and head pain in mentally unhealthy participants [14]. A study conducted in Thailand investigated GHQ-12 aspects with the pattern and quality of sleep, and it showed significantly a higher occurrence of psychological conditions such as depression and anxiety in participants with poor sleep quality [16].

In the Middle East, one case control study utilized GHQ-28 to analyze factors related to atypical chest pain [17]. Results found that atypical chest pain was an independent predictor of abnormal GHQ-28 [17]. Comorbid anxiety, depression, sleep disturbance, somatization, and social dysfunction were also prevalent among patients presented with atypical chest pain [17]. In Saudi Arabia, a population-based study has analyzed several factors related to pain chronicity [18]. It was found that the presence of medical diseases, psychological disorders, a long history of smoking, and higher scores in GHQ-12 were all considered significant predictors of self-reported chronic pain [18]. Several studies have addressed the relationship between chronic pain and psychiatric comorbidities; however, few analyzed the pattern of specific physical pain sites and patients’ psychological status. Therefore, this study was conducted in order to investigate the association between psychological elements and specific anatomical sites of chronic pain in the central region of Saudi Arabia.

## Method

### Study design and setting

This is a cross-sectional study, and the sample size collected was from January to June 2016 from Alkharj, Saudi Arabia. The details of study population and methodology were published previously [18, 19].

### Inclusion and exclusion criteria

Inclusion criteria included the following: being above 18 years of age, being a Saudi resident, and signing the consent form. Exclusion criteria included non-Saudi resident, younger than 18, or refusing to participate in the study.

### Sampling technique

A multi-stage stratified cluster sampling was used in the study. All participants were recruited from different governmental and private institutes. First, educational corporate institutes were identified and then divided into two strata: governmental and private, with a total number of 32 institutes. After that, and through cluster sampling, four public and three private institutes were selected, two out of all seven institutes were colleges. Third, each institute provided a list of respondents for its departments, where another cluster sampling took place. Finally, 1200 participants of selected clusters agreed to participate and signed the consent form. Participants were free to answer the questions without any influence from surveyors. After collecting the data, any participant who did not complete the questionnaire was excluded. That gave us a total number of 1003 of respondents.

### Material/instrument

The questionnaire had two sections. The first section was a validated General Health Questionnaire-12 (GHQ-12) for Arab population [19, 20]. The GHQ-12 is a self-reported questionnaire that consists of 12 common items, each question assessed through a four-point scale (less than usual, no more than usual, rather more than usual, much more than usual). The Likert method of scoring was used, and the total score ranged from 0 to 36 depending on respondents. The General Health Questionnaire (GHQ) is a screening tool designed to assess the respondent’s psychological status. The original GHQ comprised 60 items and contains several versions such as GHQ-1, GHQ-12, GHQ-20, GHQ-28 and GHQ-30. GHQ-12 is a shortened version and it is used extensively in medical research and primary care settings [21]. GHQ-12 can address two to three factors. The two-factor version represents psychological distress and social dysfunction [22], while the three-factor structure represents anxiety and depression, social dysfunction, and loss of confidence [23]. Moreover, for the two factors property of GHQ-12, a study done in Iran showed excellent structural characteristics, good validity and reliability GHQ-12 [20]. Another Saudi study assessed the factorial structure and psychometric properties of GHQ version 12 in non-clinical settings and revealed good measurements of the GHQ-12 three-factor model as a tool for evaluating psychological status in Saudi population [19].

The second section was about chronic pain. Chronic pain was defined as any pain that the respondents experienced for more than 3 months. In addition, an anatomical illustration was provided as a picture for respondents to locate the pain, and responses were recorded using bimodal approach.

### Data analysis

Data analysis was performed using statistical software SPSS version 26.0 for Windows. Categorical data were presented by frequency and percentage while numerical data were presented as mean and standard deviation. Simple and multiple logistic regression were used to find any association between chronic pain and items of GHQ-12. Confidence interval is 95%, and *P*-value will be significant if it is equal or less than 0.05.

### Ethical approval

Ethical approval was taken from the local Institutional Review Board, the “Committee of Scientific Research and Publication”. Written informed consent was attained from participants. All participants were notified that all data will be kept confidential and that participation is voluntary.

## Results

### GHO-12 score and pain sites categorical variable (one-way ANOVA)

We used a total GHQ score (mean = 12; SD = 5.23); as reported previously [19], to compare between eight (*n* = 8) pain sites using a categorical pain variable with the following pain sites: ‘No pain’, ‘Chest pain’, ‘Head pain’, ‘Back pain’, ‘Abdominal pain’, ‘Lower limb pain’ (thigh/leg/feet), ‘Upper limb pain’ (shoulder/arm/elbow/wrist), and ‘Neck pain’. The overall one-way ANOVA model was statistically significant (*F* = 5.279, *d* = 7, *P* < 0.0001) (Table 1).

**Table 1:** Mean difference in total GHQ-12 score by pain site/location (one-way ANOVA)

Ordinal general pain variable (8 pain sites)	*N*	Mean GHQ score	Std. deviation	Std. error
1. No pain	825	11.52	5.199	0.181
2. Chest pain	15	13.40	4.437	1.146
3. Head pain	30	14.70	5.174	0.945
4. Back pain	46	13.41	4.983	0.735
5. Abdominal pain	39	12.28	4.774	0.764
6. Lower limb pain (thigh/leg/feet)	28	11.75	5.254	0.993
7. Upper limb pain (shoulder/arm/elbow/wrist)	6	14.83	4.262	1.740
8. Neck pain	14	17.43	4.433	1.185
Total	1003	11.87	5.227	0.165
Model				
Fixed effects			5.151	0.163
Random effects				1.315

### Tukey’s post hoc analysis

Within the one-way ANOVA model above, we performed several Tukey’s post hoc multiple comparison tests (comparing the eight pain sites against each other). One post hoc analysis illustrated that compared to the ‘No pain’ site (as the reference category), the ‘Head pain’ site was statistically significant (mean difference 3.184, *P* = 0.021). The 95% confidence interval (CI) was from − 6.09 to − 0.28.

A second post hoc analysis showed the ‘Neck pain’ site was significantly different from the ‘No pain’ site (mean difference of − 5.912, *P* = 0.001). The 95% CI = (− 10.13 to − 1.70).

A third post hoc analysis showed that the ‘Neck pain’ site was statistically significant when compared with ‘Abdominal pain’ site (as the reference category). The mean difference is − 5.147, *P* = 0.030 and the 95% CI = − 10.02 to − 0.27).

And lastly, a fourth post hoc analysis illustrated that the ‘Neck pain’ site was also statistically significant when compared with the ‘Lower Limb pain’ site (as the reference category). There was a mean difference of − 5.679, *P* = 0.018 and the 95% CI = − 10.80 to − 0.56.

### Multiple linear regression analysis

We conducted a multiple linear regression analysis by regressing the total GHQ score (outcome variable) on age, gender, marital status, job type/status, diabetes status, and smoking status (Table 2). We found that the relative risk of having higher psychological distress (as indicated by a higher total GHQ score) was associated with younger age (unstandardized Beta regression coefficient = − 3.137; *P* = 0.038). Females were more likely to have higher psychological distress than their male counterparts (unstandardized Beta = 2.464, *P* = 0.003). Single/not-married people have a higher risk of psychological distress than married people (unstandardized Beta = 2.518, *P* = 0.025). Also, job type/status whether being unemployed (not working) or ‘civilian’ (civil servant/worker) was positively and significantly associated with increased probability of psychological distress—as evident by higher total GHQ score (unstandardized Beta = 1.436, *P* = 0.019). Neither diabetes status nor smoking status was significantly related to total GHQ score (Table 2).

**Table 2:** Multiple linear regression model regressing total GHQ score on ‘ordinal general chronic pain variable’, sociodemographic, and other lifestyle variables (*n* = 1019)

Total GHQ score	Unstandardized Beta (*B*)	S.E. of *B*	Sig.	Standardized *B*	95% CI for odds ratio	
					Lower	Upper
Ordinal general chronic pain variable (8 pain sites)	2.568	0.104	< 0.0001	1.136	1.249	3.656
Age	− 3.137	0.028	0.038	1.061	− 2.018	− 4.091
Gender (female)	2.464	0.492	0.003	1.137	1.498	4.430
Marital status (single/not married)	2.518	0.446	0.025	1.048	1.392	3.357
Job (not working; or civilian)	1.436	0.412	0.019	1.046	1.245	2.373
Diabetes	− 0.457	0.861	0.595	− 0.018	− 2.147	1.232
Smoking status	0.149	0.296	0.616	0.018	− 0.433	0.731

## Discussion

### Summary body sites results

The findings of the current study show that psychological well-being is adversely affected among patients with pain in different parts of the body. We also found a variation in the magnitude of psychological disorders based on the site of pain. For example, the patients with chest, back or neck pain had on an average higher risk of developing negative psychological disorders than patients with no pain. Our multiple regression analysis results found that young patients, females, unemployed, and single (or not-married) study participants were more likely to develop psychological disorders. As per our previous study, the most commonly reported site of physical pain in the Saudi population was back pain (30%), followed by abdominal pain (26%), headache (13%), knee pain (11.62%), chest pain (11.11%), and neck pain (9.09%) [18]. In this analysis, neck pain was statistically associated with higher GHQ-12 scores when compared to “no pain”, abdominal pain, and lower limb pain. Also, “head pain” achieved significantly higher GHQ-12 scores when compared to “no pain”.

### Strengths

There are several methodological issues that need to be discussed in this study. This is the first analysis that has been carried out to find an association between chronic pain and psychological distress. We also illustrated for the first time the possible psychosocial factors associated with the experience of chronic pain in an Arabian cohort, since physical and psychosocial interaction to distress varies across different nations and ethnicities [24]. This population-based study enrolled all Al-Kharj institutes using multi-stage stratified clustering technique to possibly make our sample to represent population in the central region of Saudi Arabia. In addition, we utilized GHQ-12 psychometric properties to measure psychological distress [19, 25]. Due to its high sensitivity and specificity, GHQ-12 is extensively used as an effective form of screening in primary care setting [19].

### Limitations

Since the control for chronic disease was self-reported in our study population, there is still a possibility for undiagnosed clinical comorbid syndromes such as fibromyalgia, osteoporosis, and arthritis that might alter the symptomatology and experience of psychological distress. However, GHQ-12 remains inapplicable during emergency setting. Despite the fact that GHQ predict anxiety-induced chest pain, which often presents with palpitations, elevated blood pressure, and shortness of breath related to the activation of sympathetic system, it is still insufficient to rule out chest pain of a cardiac origin [26].

### Comparison section: pain effect on social and anxiety domains

In this study, chronic pain was significantly associated with social health impairment in terms of concentration, decision-making, and day-to-day activity. This is in line with what was reported by Moulin et al. who found that half of patients with chronic pain and active pain symptoms had poor contact with their families, which affects the desirability to attend social events [27]. Another study stated that neuropathic pain could strongly affect patients’ social and mental health [28].

The results of this study indicated that patients with chronic pain could significantly develop depressive disorders. In fact, it has been proved that chronic pain increases the chances of developing depression by two- to fivefold [29]. Moreover, patients complaining of multiple pain sites are more likely to have depressive disorders [29]. A study done in a tertiary hospital in Saudi Arabia revealed that 71% of patients with chronic pain also suffer from depression, which is higher than other similar studies [30]. Bair et al. revealed in a systematic review that the prevalence of depression in patients with chronic pain can reach up to 46% in primary healthcare studies and up to 22% in population studies [10]. Another recent study on 10 million patients who suffered from chronic pain found that 23% developed depressive symptoms [31]. In the Saudi population, it has been found that being middle-aged, poor financial status, and severe chronic pain were the risk factors for co-occurrence of chronic pain with depression [30].

### Comparison sections: site of pain and psychological distress using GHQ-12 version

Results of our study are in line with several studies discussing the close association between specific pain sites and psychological distress measured using GHQ-12 [14, 32–35]. Patients with chronic widespread pain were at a higher risk of having mental disorder, three times that of the healthy group [32].

Zhaojia et al. showed that the subjective report of back pain was significantly higher among mentally unhealthy Visual Display Terminal (VDT) workers [33]. In the UK, GHQ-12 scores could predict the prevalence of back pain among workers from five different jobs including mail carriers, cashiers, shelf stackers, production line worker, and nurses. The prevalence rate ratio increased from 1 for GHQ-12 scores of 12–20, 1.5 for GHQ-12 scores of 21–23, and substantially reached 2.3 for GHQ-12 scores of 24–48 [34]. Likewise, the results of GHQ-12 in Germany revealed that mentally distressed teachers complain of neck and back pains (78%) more than their healthy counterparts (64%). Headache was also experienced in 58% among mentally impaired teachers compared to 39% among healthy group [35]. Similarly, mentally unhealthy Japanese teachers, including both male and female with higher GHQ-12 scores, were found to subjectively report pain more in certain body sites with neck–shoulder pain, eye pain, lower-back pain, and headache being the most commonly reported. After multivariate adjustment, mentally unhealthy male had significantly higher odds ratio for pain reported in eyes (1.55), upper extremities (1.50), head (1.46), upper back (1.46), and neck–shoulder (1.27) [14]. Mentally unhealthy females reported pain more in the lower extremities with an odds ratio of 1.440 compared to male (0.9). Gender difference in the subjective experience of pain sites might be attributed to the behavioral nature of female teachers who stand most of working hours bearing their weight in the feet [14]. Moreover, previous international and local data assume that knee pain is more prevalent among females, which might be attributed to the structural difference between the sexes in the bony architecture and cartilaginous structure [18, 36]. One plausible and reasonable explanation for females being at higher risk of depression than males could be that females are more persuaded to visit health care facilities and hence are more likely to be diagnosed with psychological problems than males. In addition, females are also prone to stresses of life due to their reproductive life span and course of life [37, 38]. Additionally, increased survival among females can also make them vulnerable to develop negative psychological disorders than their counterparts. This is because increased life expectancy may increase the exposure of females to risk factors, thereby negative psychological disorders. Variations of the reported pain sites among mentally distressed cases could be explained by different study settings and variations in the culture and population of different countries.

Besides, we found that younger people are at higher risk of developing psychological disorders than older people. The existing premise also reveals that the burden of depression declines with age, thereby proving an inverse association between age and psychological disorders. Even there are gender differences by age, meaning that the burden of anxiety and depression reduces among females as they grow older thus further strengthening the relationship between age and psychological disorders [39]. Lastly, our findings from the adjusted analysis showed that single or unmarried people were at higher risk of developing negative psychological disorders than married people. This finding is consistent with other findings in the literature that have found a similar relationship between marital status and psychological disorders [40, 41]. These findings also confirm that being married is not protective only for Western people, but also for those living in other parts of the world [42]. Findings from a meta-analysis also revealed that being married increases the risk of depression mainly in the older age [43].

### Implications of the study

Both pain and psychological distress are considered major predictors of well-being. The relationship between chronic pain and mental illness, especially depressive disorders, was previously hypothesized to be bidirectional. It is a well-recognized phenomenon that prolonged, unresolved mental distress might unconsciously translate into physical symptoms [44]. Wei et al. reported that 19.7% of patients diagnosed with depression, 13.7% diagnosed with anxiety, 15.2% diagnosed with somatic symptom disorder had headache as the initial presenting symptom [45]. A recently conducted systematic review showed higher rate of depression and anxiety among subjects with neck pain [46]. The prevalence of somatoform disorder ranges from 10.7 to 26.8% in young populations complaining of medically unexplained symptoms [47]. On the other hand, chronic disabling pain could restrict patients from performing simple daily life activities leading to individual dependence. As a result, more depressive symptoms would develop [44]. Such a complexity in the presentation of chronic pain often results in diagnostic, therapeutic dilemmas, leading to unnecessary expensive investigations and visits. Furthermore, the burden of psychiatric distress exacerbates more among patients when their chronic pain is perceived as difficult by professional physicians.

### Recommendations

It is necessary for all professional physicians to follow the bio–psycho–social model while approaching patients presenting with a chronic pain disorder. The implementation of GHQ-12 as a screening tool in the primary care settings would effectively help detect and treat underlying psychiatric illness earlier to achieve a better quality of living perception of physical functioning.

### Conclusion

Our study found a significant association between chronic pain and social dysfunction or depressive disorders. Moreover, high GHQ-12 scores were associated with chronic neck pain when compared to “no pain”, abdominal pain, and lower limb pain. In addition, patients with chronic neck pain had higher GHQ-12 scores when compared to “no pain”. These findings have important implications in terms of not only screening such people with chronic pain for negative psychological disorders, but also exploring the underlying reasons and providing them timely and effective therapeutic support to prevent them from developing negative psychological problems. Furthermore, diagnosis, screening, and treatment must be regularly integrated into the daily care of such patients with chronic pain in different body parts. In addition, to prevent them from having negative psychological results and their long-term complications, the government of Saudi Arabia needs to concentrate on females, young and unmarried individuals as high-risk populations and devise some high-risk strategies with a diversion of resources to these vulnerable populations of Saudi Arabia. Well-designed longitudinal studies, primarily in Saudi Arabia, are needed in the future to research the cause and impact of poor mental health and chronic pain. In addition, further studies are also needed to make causal inferences and to explore the relationship between poor mental health and chronic pain in the Saudi population, as well as important factors of this association.

## Data Availability

The dataset generated and analyzed to support the findings of this study was provided by Prince Sattam bin Abdulaziz University under license and is not publicly available. Access to these data will be considered by the author upon request, with permission of Prince Sattam bin Abdulaziz University.
